# Blood-Brain Barrier Opening in Behaving Non-Human Primates via Focused Ultrasound with Systemically Administered Microbubbles

**DOI:** 10.1038/srep15076

**Published:** 2015-10-26

**Authors:** Matthew E. Downs, Amanda Buch, Maria Eleni Karakatsani, Elisa E. Konofagou, Vincent P. Ferrera

**Affiliations:** 1 Department of Biomedical Engineering, Columbia University, New York, New York, United States of America; 2 Department of Radiology, Columbia University, New York, New York, United States of America; 3 Department of Neuroscience, Columbia University, New York, New York, United States of America

## Abstract

Over the past fifteen years, focused ultrasound coupled with intravenously administered microbubbles (FUS) has been proven an effective, non-invasive technique to open the blood-brain barrier (BBB) *in vivo*. Here we show that FUS can safely and effectively open the BBB at the basal ganglia and thalamus in alert non-human primates (NHP) while they perform a behavioral task. The BBB was successfully opened in 89% of cases at the targeted brain regions of alert NHP with an average volume of opening 28% larger than prior anesthetized FUS procedures. Safety (lack of edema or microhemorrhage) of FUS was also improved during alert compared to anesthetized procedures. No physiological effects (change in heart rate, motor evoked potentials) were observed during any of the procedures. Furthermore, the application of FUS did not disrupt reaching behavior, but in fact improved performance by decreasing reaction times by 23 ms, and significantly decreasing touch error by 0.76 mm on average.

The blood-brain barrier (BBB) is a protective system that maintains homeostasis of the brain^1^. While this protection is essential to prevent noxious or infectious agents from reaching the brain, the BBB also prevents almost all large-molecule and 99% of currently available small-molecule (>400 Da) drugs from accessing the brain^2^. Focused ultrasound (FUS) combined with systemically administered microbubbles (MB) has been shown as an effective, non-invasive technique to open the BBB in multiple *in vivo* models including non-human primates (NHP)^3^,^4^,^5^,^6^. The application of FUS with MB is multifaceted and can be utilized both in the clinical and laboratory settings. In the clinic, it can be used to facilitate drug delivery for the treatment of neurological diseases or disorders such as Parkinson’s and Alzheimer’s disease, which currently do not have targeted, or non-invasive treatment options^7^,^8^,^9^. Within the laboratory, the procedure can be used for targeted drug delivery for neuromodulation experiments, or evaluation of novel drugs that cannot cross the intact BBB. The procedure has already been successful for the transport of various molecules such as doxorubicin and neurturin across the BBB, for treatment of tumors and to facilitate neuroprotection^10^,^11^. Currently, FUS has only been shown to be safe and effective in anesthetized animal models^12^,^13^,^14^. The anesthesia requirement may not be ideal in a future clinical setting. In addition, anesthesia may affect behavioral experiments in a laboratory setting^15^. For the full potential of this technique to be utilized in both the clinical and laboratory settings, it must be shown to be safe and effective with alert subjects.

One major difference in physiologic conditions when performing the procedure on an anesthetized rather than alert subject lies in the effects of the anesthetic on the vascular system. Isoflurane, a common anesthetic used for surgical procedures and anesthetized experiments in animals, causes vasodilation and a decrease of vascular resistance in cerebral vasculature^16^,^17^. Average cerebral capillary diameters of NHP are typically 4–8 μm, but under isoflurane anesthesia vasodilation occurs which can lead to an increase in cerebral blood flow^18^,^19^,^20^. One prominent theory about how FUS causes BBB openings postulates that MB in the vasculature oscillate due to the FUS exposure. These MB oscillations physically disrupt the endothelial cells that comprise the BBB^21^,^22^,^23^,^24^. The two main types of bubble activity reported during FUS procedures are stable and inertial cavitation^25^. Stable cavitation consists of both harmonic and ultraharmonic oscillations of the MB and is the dominant mechanism for BBB opening when the MB diameters are similar to the vessel diameter^21^. Inertial cavitation is caused when the MB collapse, generating high energy jets that can damage the vasculature. Inertial cavitation is the dominant mechanism when the MB diameters are smaller than the vessel diameter^21^,^23^,^26^. As our group utilizes MB with an average diameter of 4–5 μm, the smaller vessel size during alert FUS procedures due to the lack of isoflurane could change the occurrence of stable and inertial cavitation compared with anesthetized procedures. This change in cavitation could translate to a difference in BBB opening volume as stable cavitation has been correlated with smaller, safe opening volumes while inertial cavitation has been correlated with larger BBB opening volumes that have the potential to cause damage (edema, erythrocyte extravasation)^27^,^28^,^29^. The smaller diameter of the vasculature would also increase the overall force the MB apply on the endothelial cells from stable oscillations, potentially causing an increase in damage to the vasculature^30^,^31^. Aside from vessel diameter, cerebral blood flow and persistence time of MB in the vasculatures are also affected by the use of isoflurane mixed with pure oxygen as an anesthetic^19^,^20^,^32^,^33^. Changes to those parameters would affect the dosage of the MB reaching the area targeted by the FUS. For FUS, isoflurane anesthesia generates unfavorable conditions, the removal of which could have an effect on the safety of the tissue in the targeted region and success of achieving BBB opening.

In this study, we show that FUS can be a safe and effective technique to open the BBB in alert NHP. The NHP were trained to perform a visually guided reaching task to receive fluid rewards. The NHP were head-fixated in a primate chair allowing for targeted FUS application while they simultaneously performed a behavioral task designed to test motor control and motivation. The caudate and putamen regions of the basal ganglia as well as the thalamus were targeted by the FUS as these regions are greatly affected by Parkinson’s disease, which currently does not have a reliable long-term treatment solution^34^,^35^,^36^. These regions are also implicated in memory, voluntary motor control, goal-directed action and decision making^37^,^38^,^39^. The behavioral task utilized the well-established reward magnitude bias paradigm and measured visual perception, motivation and motor control to test the function of the targeted regions during application of the FUS technique^40^,^41^. T2-weighted and susceptibility-weighted MRI images were used to investigate the safety of the technique while contrast enhanced 3D T1-weighted sequences were used to verify BBB opening. The results of behavioral testing, along with the MRI findings demonstrate that the FUS procedure for BBB opening can be safe and effective in alert, behaving subjects.

## Results

### Safety

Initial trials of the FUS procedure on a lightly sedated (single 5 mg/kg dose of ketamine) NHP (NHP B) did not elicit any autonomic changes. Heart rate and blood pressure remained consistent throughout the procedure (136 beats per minute, 68 mean arterial pressure). No sudden limb, jaw or eye movement, nor pupil dilations were observed by researchers or the observing New York State Psychiatric Institute (NYSPI) veterinary staff. After the procedure, NHP B recovered normally from light anesthesia and returned to routine activities (playing, eating, and drinking). T2-weighted MRI and susceptibility weighted image (SWI) scans did not show any abnormal hyper- or hypointense voxels in the targeted regions signifying no edema or MRI-detectable microhemorrhage had been caused by the procedure.

**Figure 1:**
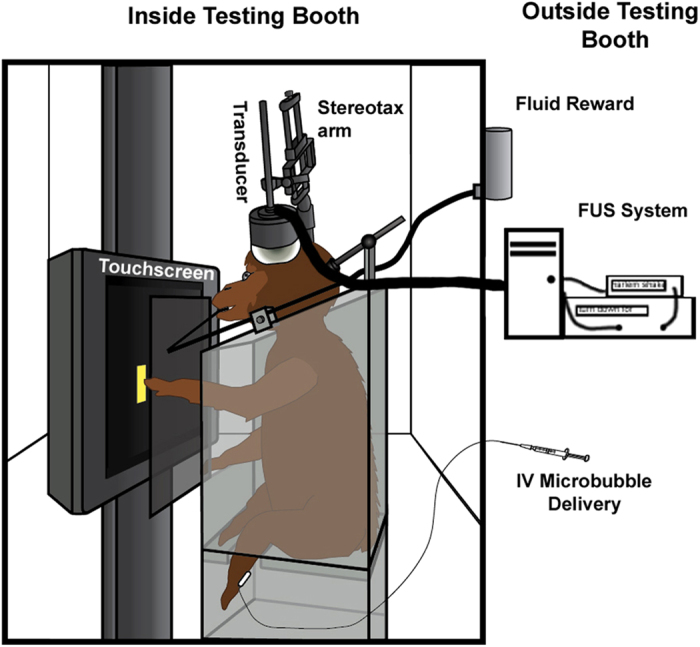
Alert FUS Procedure Setup. The NHP is placed into a primate work chair and is head fixated. This position allows free movement of their arms to respond with the ipsilateral arm as the stimuli from the behavioral task displayed on the touch monitor. The transducer, EMG leads and pulse oximeter were positioned on the scalp of the NHP. The experiment was run external to the booth allowing the NHP to complete the behavioral task unaware when the FUS technique was applied.

**Figure 2:**
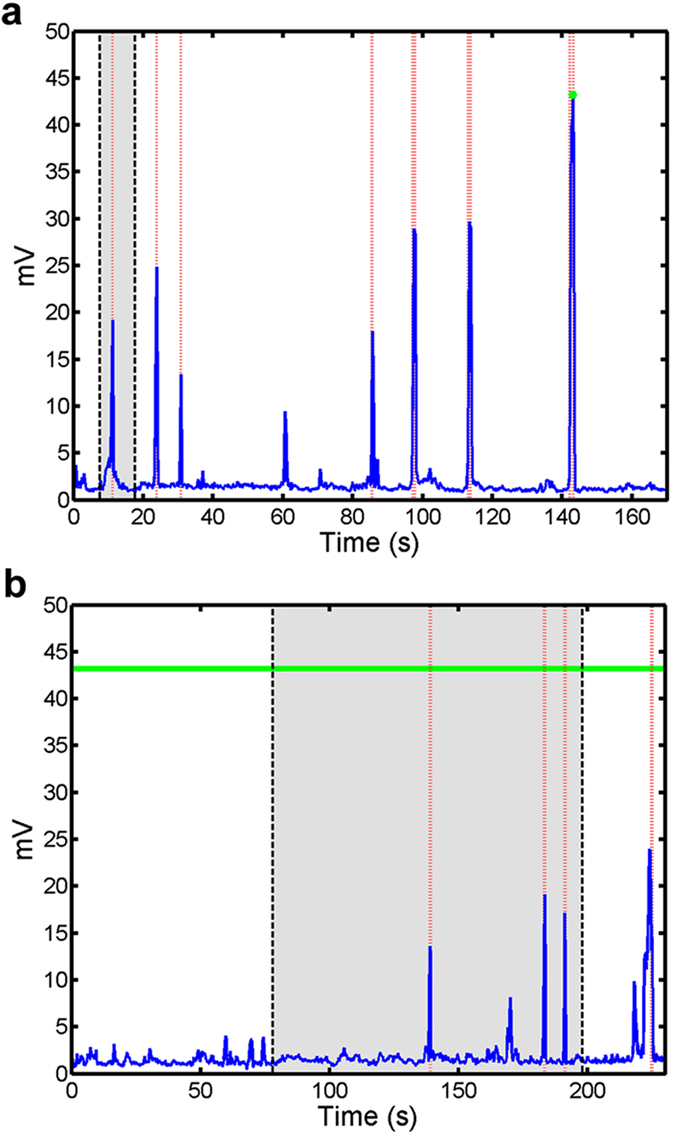
EMG Recordings. EMG signals were recorded from the temporal muscle contralateral to the FUS application. The grey region denotes when either the control (**a**) or the full sonication (**b**) with the transducer was being applied. Red dashed vertical lines indicate detected muscle activation. The horizontal green bar indicates the maximum signal recorded during the control period (indicated by the green dot) and subsequently used as the threshold to detect abnormal or large signals that occurred during the application of the FUS technique. Only recordings from NHP Z are shown, but NHP A showed similar responses. There was no abnormal or large muscle activity triggered by the alert FUS procedure.

Having verified the preliminary safety of the procedure in a lightly sedated NHP, the FUS technique was applied to fully alert NHP. The full setup for the alert FUS technique along with behavioral testing can be seen in Fig. 1. As with the lightly sedated experiment, application of the FUS technique did not cause any macroscopic motor effects (sudden limb, body or eye movement) nor did it elicit signs of pain (facial grimace) from either NHP, regardless of the targeted brain region. Heart rate remained consistent throughout the FUS technique and within the normal range for fascicularis macaques (mean ± s.d.: NHP A: 152.4 ± 1.2 BPM, NHP Z: 179 ± 8.9 BPM). The larger variation in the heart rate for NHP Z arose from motion artifacts in the SpO_2_ signal, as he was generally more active while he worked. Neither of the NHP exhibited an abrupt change in heart rate at the onset or during the application of the FUS technique. EMG recordings on the temporalis muscle only detected normal jaw and mouth movements (licking reward tube, smacking lips) during the procedures (Fig. 2). There were no abnormal or large EMG signals detected either during or after the FUS procedure that surpassed the recorded activity during the control.

**Figure 3:**
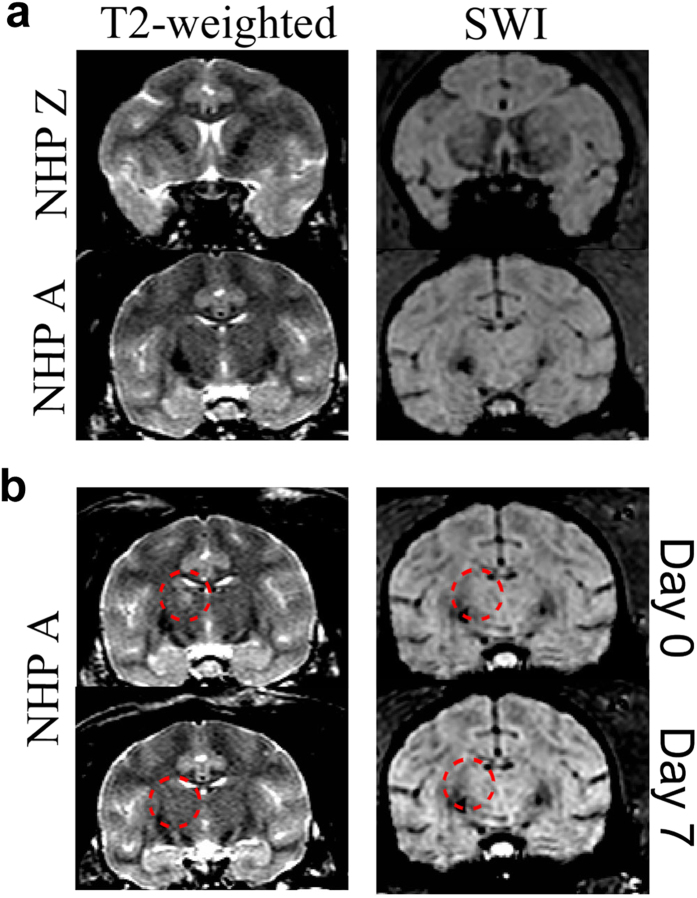
MRI Safety Verification. T2-weighted and SWI sequences were acquired to verify the safety of the alert FUS procedures. (**a**) shows typical cases of both T2-weighted and SWI scans of the targeted regions. There were no abnormal hyper- or hypointense voxels present in any of the targeted regions (caudate, putamen or thalamus). (**b**) shows the one case for NHP A when there were hyperintense voxels in the targeted region in the T2-weighted scan, denoted by the red dashed circle. This area of hyperintense voxels could indicate the presence of edema. By day 7 the hyperintense voxels were absent. Neither on day 0 nor 7 were any hyper or hypointense voxels detected in the target regions of the SWI scans.

The majority of the T2-weighted and SWI images did not show any abnormal hyper- or hypointense voxels for either NHP Z or A (Fig. 3a). In one case, NHP A exhibited unusual hyperintense voxels on a T2-weighted MRI scan on day 0 after an alert FUS procedure (i.e. the day of the procedure, Fig. 3b). This suggests the potential for transient edema in NHP A when targeting the caudate. The hyperintense voxels were not present on day 7. No hyper- or hypointense voxels were detected in the region of possible edema on SWI scans on either day 0 or day 7. These results show that the FUS technique has some possibility to cause reversible edema without the presence of MRI-detectable microhemorrhaging in alert NHP. By comparison, during 13 anesthetized procedures, NHP A had 3 cases of hyperintense voxels on the T2-weighted scans on day 0. Similar to the alert experiments, the hyperintense voxels were not present on day 7. Histological evaluation was not conducted on either NHP as they were required for further experiments.

### BBB Opening

**Figure 4:**
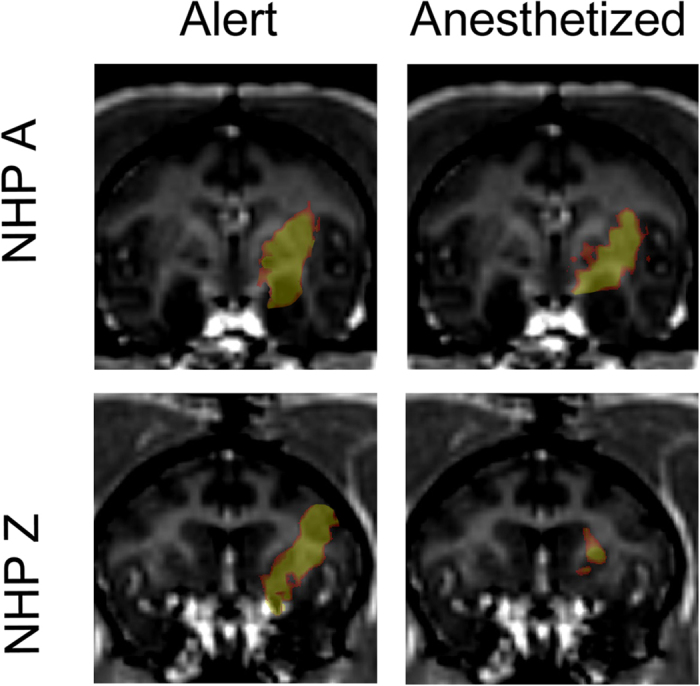
BBB Opening Verification. Contrast enhanced T1- weighted sequences were acquired to verify the opening of the BBB. The transparent yellow regions indicate the BBB opening volume. Typical cases of BBB opening for the alert FUS procedures are in the left column, while typical results from anesthetized FUS procedures are seen on the right column.

**Figure 5:**
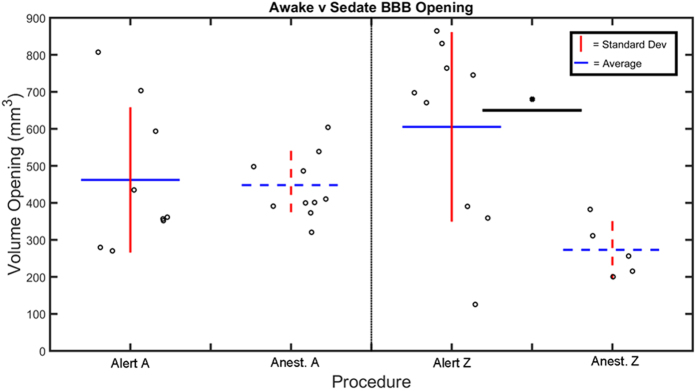
Alert vs Anesthetized BBB Volume. The average volume of BBB opening from the alert and anesthetized FUS technique were compared per animal (A or Z). The mean is indicated with a blue horizontal bar while the standard deviation is indicated by a red vertical bar. The black circles are individual BBB opening cases. The dashed bars indicate anesthetized experiments. Only NHP Z had a non-significant increase in the volume of BBB opening for alert FUS procedures over anesthetized (2-sided WRS test, p = 0.029) indicated by the black horizontal bar with a dot.

**Figure 6:**
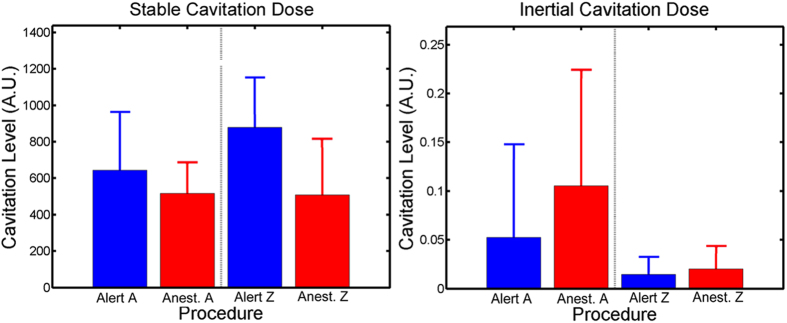
Cavitation Doses. During both the alert and anesthetized FUS procedures, the passive cavitation detection recorded the signals emitted from the MB in the focal area of the transducer. Blue bars indicate average cavitation dosage and 95% confidence interval of the mean for alert FUS procedures while red indicates average cavitation dosage 95% confidence interval of the mean for anesthetized FUS procedures. (**a**) shows the average stable cavitation doses while (**b**) shows the average inertial cavitation dosages. For both NHP there was a non-significant increase in stable cavitation doses between the alert and the anesthetized FUS procedures. The inverse occurred with the inertial cavitation dose with smaller doses detected during the alert compared to the anesthetized FUS procedures.

Post contrast T1-weighted MRI sequences verified BBB opening for the majority of the alert FUS procedures on NHP A & Z. Typical cases of BBB opening are indicated by a transparent yellow color map overlaid on the T1-weighted MRI scans shown in Fig. 4. The contrast enhanced areas cover the targeted putamen region for NHP A and NHP Z. Successful BBB openings were obtained in 8/9 (NHP A) and 9/10 (NHP Z) alert FUS procedures. Figure 5 shows NHP A exhibiting smaller BBB openings on average (mean ± s.d.: 462.0 ± 193.4 mm^3^) than NHP Z (mean ± s.d.: 605.3 ± 253.2 mm^3^) after alert FUS procedures. Success rate of BBB opening and the average of the BBB opening volumes per location and NHP are listed in Table 1. Both NHP showed on average 28% larger BBB opening volumes for the alert compared to anesthetized FUS procedures. Only NHP Z exhibited a significantly larger BBB opening volume for the alert compared to anesthetized procedures (2-sided WRS test, p = 0.029). Figure 6 shows a non-significant increase in the detected stable cavitation dose from the passive cavitation detection (PCD) for the alert procedures over the anesthetized procedures for both NHP (2-sided WRS test, p = 0.13 and p = 0.46 respectively). There was also a non-significant decrease in inertial cavitation dose between the alert and anesthetized procedures (2-sided WRS test, p = 0.70 and p = 0.57 respectively).

### Behavioral Results

**Figure 7:**
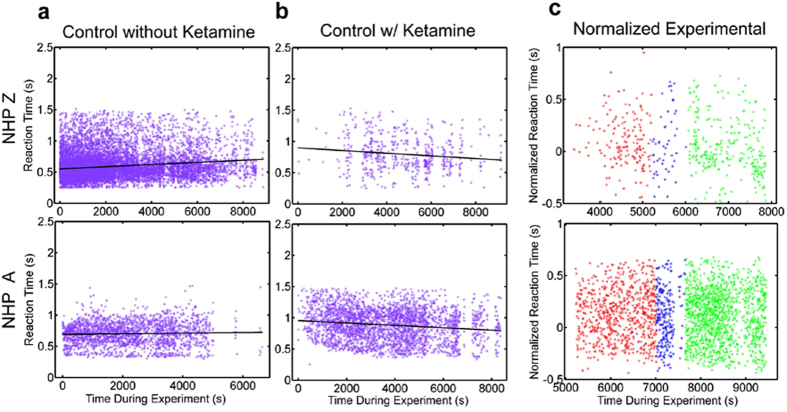
Raw Reaction Time Data. Reaction times for individual trials as a function of time during the experiment. For (**a**) and (**b**) magenta dots indicate individual data points and the black line is the linear regression fit. (**a**) shows the control data collected on days when ketamine was not administered. (**b**) shows the control data collected on days when ketamine was administered to mimic the parameters used on experimental days. The regression fit for the ketamine control data was used to normalize the experimental data to remove the effects of ketamine on the behavioral results during experimental days. (**c**) shows the normalized experimental data. Red dots indicate trials completed before applying the FUS technique, blue indicates trials completed during the FUS technique and green indicates trials completed after applying the FUS technique.

To determine if the FUS procedure affected visuomotor behavior or motivation, the NHP were trained to perform a visually guided reaching task with differential reward. The RMB task completed by the NHP during the FUS technique was utilized as a more sensitive evaluation of the potential side effects of the technique on the central nervous system of the NHP. The behavioral variables recorded were reaction time (RT) and touch error (TE). Individual responses to the task for the control and experimental days are shown in Fig. 7. On days when the FUS technique was administered, the NHP were given a low dose of ketamine for placement of the IV catheter prior to behavioral testing. This resulted in a slight anesthetic effect that was observed in the behavioral results (Fig. 7b). The control data after ketamine show elevated RTs at the beginning of each day’s behavioral testing that decrease over the duration of the session (Fig. 7b). This decrease was not observed on days when the animal performed the task without any prior ketamine (Fig. 7a). Thus, the RT decrease along the entire duration of the behavioral task can be attributed to the lingering effects of the ketamine. As a control, on some days, the same low dose of ketamine used when placing the IV catheter was administered prior to behavioral testing, but the FUS technique was not applied. Linear regression was performed on the ketamine control data and used to subtract out the effects of ketamine from the experimental data (days when the FUS technique was applied, Fig. 7c). This normalization was performed for RT and TE.

**Figure 8:**
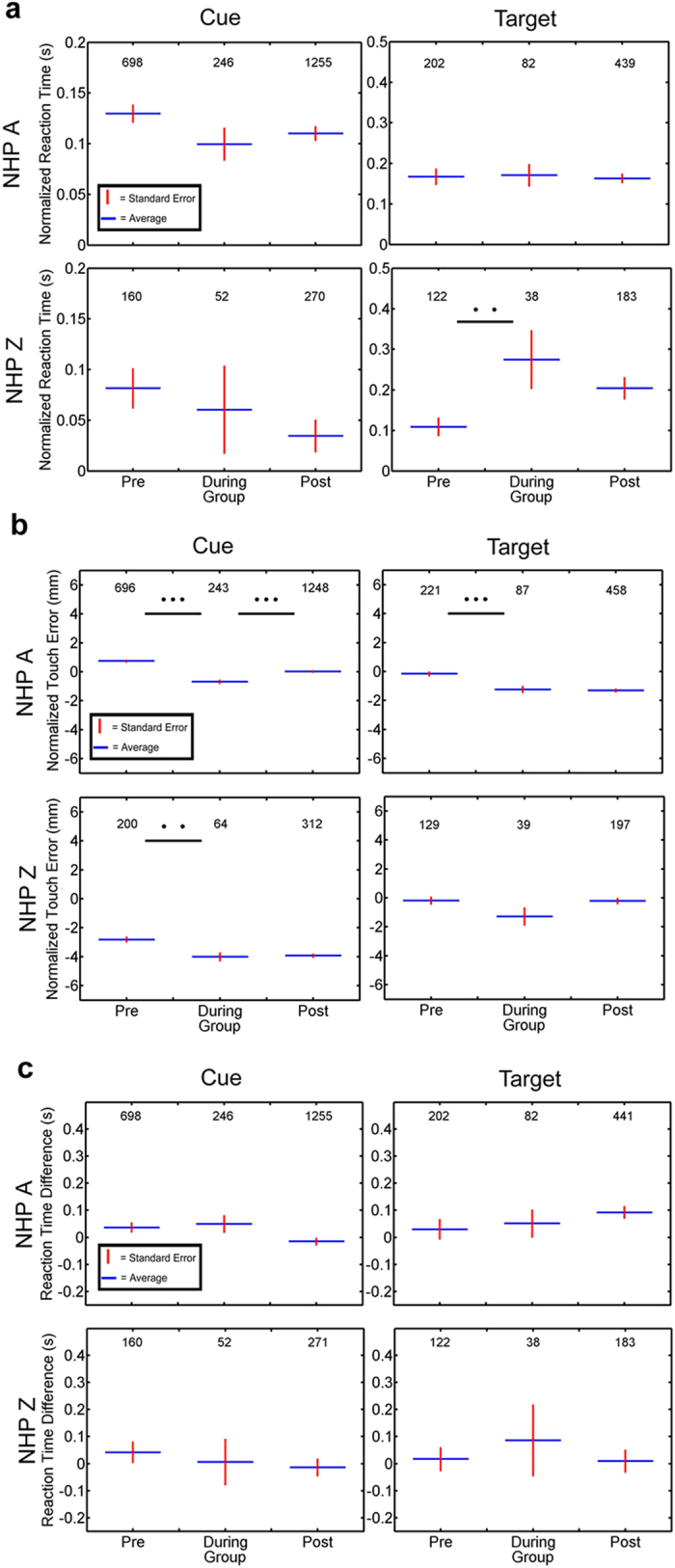
Behavioral Results. The responses to the behavioral task were measured in reaction time and touch error. The numbers at the top of the Figure indicate the n value (number of completed trials) for each group. (**a**) shows the average reaction time to the cue and target for each group (pre, during and post FUS procedure). The horizontal blue bar indicates the average reaction time for each group while the red vertical bar indicates the standard error. There is a small non-significant decrease across the groups for both NHP reacting to the cue (2-sided student’s t-test, p > 0.05). NHP A exhibits this decrease in reaction time across groups when responding to the target, but NHP Z shows a significant increase in reaction time to the target (2-sided student’s t-test, p = 0.013). (**b**) shows the average touch error for each group. Blue horizontal bars indicate the average touch error while red vertical bars indicate the standard error. For both NHP there was a significant decrease in touch error between the pre- and the during group in response to the cue (2-sided student’s t-test, p < 0.01). Only NHP A also exhibited a significant difference in touch error between the pre- and during groups to the target. (**c**) shows the difference in reaction time between the high and the low reward. Horizontal blue bars indicate the average difference in reaction time (high-low) while the red vertical bars indicate standard error.

On each trial of the behavioral task, the NHP first touched a cue stimulus and then touched a target presented four cm away from the cue (see Methods). One benchmark to determine if the FUS procedure had an effect on the NHP while they conducted the behavioral task was to evaluate their RT to the cue and target stimuli. Each session’s data were divided into three groups: pre (before sonication), during sonication and post (after sonication). The average RTs to the cue and target are shown in Fig. 8a. NHP A did not show significant variation in mean RT between groups, nor significant difference between the pre- and during or post- and during groups to the cue and target stimuli (1-way ANOVA, p = 0.138 and p = 0.960 for cue and target respectively, 2-sided student’s t-test, p = 0.092 and p = 0.091 for cue groups respectively, p = 0.911 and p = 0.856 for target groups respectively). NHP Z did not show any significant difference across groups for the cue stimulus, but did show a significant increase between the pre-and during groups to the target (1-way ANOVA, p = 0.203 and p = 0.012 for cue and target respectively, 2-sided student’s t-test, p = 0.619 and p = 0.070 for cue groups respectively, p = 0.005 for target group). This increase persisted into the post-sonication period, which was not significantly different from the during group (2-sided student’s t-test, p = 0.088). Overall, in three out of the four cases there was a slight decrease in reaction time after the application of the FUS procedure.

While variations in RT assess the speed of the visuomotor response, touch error (TE) can be used to detect variations in spatial accuracy of the reaching movement^42^,^43^. Touch error was determined as the distance between the center of the stimulus (cue or target) and the first point where the NHP contacted the touchscreen monitor. NHP were not differentially rewarded based on accuracy. Figure 8b shows that for NHP A there was a significant decrease in TE to the cue between the pre- and during groups, as well as between pre- and post groups (2-sided student’s t-test, p < 0.0001, p < 0.0001 respectively). This significant decrease in TE was also observed with the response of NHP A to the target stimuli (2-sided student’s t-test, p = 0.0003, p < 0.0001, respectively). Similarly, there was a significant decrease in TE for NHP Z responding to the cue stimuli (2-sided student’s t-test, p = 0.003).

Our behavioral task included a reward bias; on some trials, the NHP received 5 drops of water as a reward for correct performance. On other trials, the reward was 1 drop. A visual cue (the orientation of the cue and target stimuli) signaled the reward size. The reward value for each trial was selected randomly so the NHP could not predict which stimulus would appear prior to the start of the trial. Once the cue appeared, it informed the NHP of the reward size on that trial. NHP are often biased to respond faster and more accurately on trials with larger rewards^40^. Hence, the reward bias can help to determine if motivation was affected by the FUS procedure. For both NHP there was no significant variation across all groups nor a significant change in RT difference between individual groups (1-way ANOVA, p > 0.05 for both NHP, 2-sided student’s t-test, p > 0.05 with all comparisons for both NHP, Fig. 8c). This behavioral task has been previously employed in our lab and successfully elicited specific motivational responses to the high/low reward, but these results indicate that this did not occur during these experiments^13^. This lack of reward bias was also absent for the control data sets, and thus was not caused by the FUS technique.

## Discussion

In this study, we showed the FUS technique is a safe and effective procedure on alert NHP using MRI verification, physiological recordings and behavioral assessment. An 86% success rate for opening of the BBB at the targeted regions was achieved and the average volume of the BBB openings in alert NHP was 28% larger than openings achieved with anesthetized FUS procedures in the same NHP. The increase in BBB opening volume could be due to several factors. For example, the dosage of MB that reached the target area could have been larger during alert than anesthetized experiments. Prior studies have shown that oxygen increases the decay rate of MB in the bloodstream by approximately a factor of three^33^. As oxygen was mixed with 1.1–1.5% isoflurane during the anesthetized experiments, the MB would have a shorter circulation time. This would decrease the dosage of MB reaching the target site, and also decrease the overall PCD signal detected during the anesthetized experiments. We did find an increase in the stable cavitation dose in alert subjects, which could be attributed to the higher MB dose. However, we saw a decrease in the inertial cavitation dosage. This finding agrees with prior studies where the diameter of the vessel has an effect on the behavior of the MB inside the focal area of the transducer. When the vessel diameter is larger than the MB size, inertial cavitation is the dominant mechanism for BBB opening, and conversely when the diameter of the vessel and the MB size are comparable, stable cavitation becomes the dominant mechanism^21^. As no isoflurane was used during the alert FUS procedures, the vessels would not have been dilated, retaining their average size of ~5 μm, which is comparable to the 4–5 μm diameter MB used for the experiments^18^. This would support the finding of an increased stable cavitation dosage and a decreased inertial cavitation dosage during the alert compared to the anesthetized FUS procedures. Another factor that could have potentially altered the MB dosage is the cerebral blood flow (CBF). High doses of isoflurane have been correlated with higher CBF, which would produce larger PCD signals as more MB would be passing through the focal region during each ultrasound pulse. This increase in CBF was previously observed at isoflurane doses >1.6%^20^. For our anesthetized experiments, NHP were only initially dosed with 2% isoflurane for placement into the stereotax before being reduced to 1.1–1.5% for the duration of the experiment. Thus, in our previous anesthetized FUS procedures, we might not have reached the level of isoflurane needed to increase the CBF. The increase in stable cavitation dosage during FUS procedures with alert NHP could be attributed to a larger dose of MB, as circulation time was not reduced, as well as smaller vessel diameters that were comparable to MB diameter. Although these results are non-significant, they suggest a trend that alert FUS procedures are safer and more effective at opening the BBB.

BBB opening was safely achieved (as assessed by T2-weighted MRI and SWI scans) for the majority (17/19) of the alert experiments with only one case of potential edema in NHP A (out of 9 procedures in that animal), which resolved within a week. This is a decrease in the occurrence of hyperintense voxels appearing in the targeted region for NHP A compared to anesthetized experiments when it occurred 3 times in 13 procedures. These findings are concordant with the non-significant decrease in inertial cavitation dosage recorded during the alert FUS procedures. Prior studies have shown a correlation between inertial cavitation with edema and red blood cell extravasation^28^,^29^. The inertial cavitation dosage was lower in the alert FUS procedures, which could explain the less frequent occurrence of edema. Overall, our results show that the alert FUS procedure elicited fewer cases of potential edema on average than prior anesthetized studies.

Importantly, the procedure did not elicit any gross negative physiological reactions (ballistic motor activity, pain grimace) while the NHP were completing the behavioral task. Their heart rate remained consistent before, during and after the application of the FUS technique. EMG data also revealed no abnormal muscle activity in the local area of the transducer during the procedure. This lack of abnormal EMG signals shows that the FUS technique does not elicit muscle activity in the local area of application.

The FUS procedure was administered during ongoing performance of a self-paced visuomotor reaching task. There was no disruption in the NHP’s behavior once the task had begun. The NHP continued to initiate trials at the same rate before, during and after FUS administration. A small, non-significant decrease in reaction time to the cue stimuli was observed after the application of the FUS technique. The procedure also significantly decreased touch error by reducing the average touch error between the pre- and during group for both the cue and target stimuli for NHP A. Another FUS technique, transcranial-FUS (tFUS), is currently being investigated as an alternative to transcranial magnetic stimulation and has been shown to successfully elicit neuromodulation in partially sedate and alert NHP and humans^44^,^45^. Although tFUS utilizes different transducer parameters (pressure, pulse repetition frequency, duration of sonication) than the FUS BBB opening technique, our results show the FUS technique targeting the basal ganglia and thalamus has the potential for small beneficial effects improving the accuracy of the NHP selecting the targets present on the screen.

One of the major benefits of this technique is the lack of anesthesia required. Introduction of anesthesia into a procedure increases the potential for adverse side effects^47^,^48^,^49^. Prior studies using mannitol to open the BBB elicited seizures and thus anesthesia was required^50^. Our results did not indicate the presence of seizures nor other adverse effects while opening the BBB in alert subjects. The only use of anesthesia during our study was a low dose of ketamine required for the placement of the IV catheter, which in clinical patients would not be necessary. The lack of anesthesia also greatly reduces the overall procedure time since there are no induction or recovery periods.

This initial feasibility and safety study verifies the FUS technique is successful at opening the BBB in alert NHP without eliciting gross adverse physiologic reactions during or after the procedure. The data also showed a non-significant increase in safety and BBB opening volume compared with anesthetized procedures as well as a potential beneficial decrease in reaction time and an increase in reaching accuracy. This technique will continue to be explored as a powerful tool in the lab as it transitions to a therapeutic technique in the clinic.

## Methods

### Animal Husbandry

All NHP procedures described herein were carried out in accordance with the approved guidelines of the Institutional Animal Care and Use Committees (IACUC) of Columbia University and the NYSPI. Two adult male Macaca Fascicularis (NHP: Z, A) were used for the majority of the experiments (Ages: 14, 18 years old; weights: 5.3, 5.6 kg) and one Macaca Mulatta (NHP B, 21 years old, 10.1 kg). All NHP were housed in a 3 ft^3^ Erwin-Steffes Enhanced Environmental Housing System (Primate Products Inc., Immokalee, FL, USA) with a 12 hour light/dark cycle. They were provided weekly access to play cages (total area 3 ft^2^ × 7 ft) and various enrichment toys within their home cage. They were fed a constant ration of vitamin enriched dry primate biscuits and given 1L of water on days when they were not tested behaviorally. On testing days, the NHP performed a behavioral task for fluid reward until they were satiated. On days when the behavioral session was completed, they were given a fruit treat. Both NHP A & Z underwent IACUC approved sterile surgical procedures for a head post implantation allowing head fixation during the behavioral testing and FUS procedure.

### FUS Procedure

MB used in the FUS procedures were fabricated in house and size isolated for a mean diameter of 4–5 μm^46^. A 500-kHz center frequency focused ultrasound transducer with a built-in bladder system was used for all FUS procedures (H-107, Sonic Concepts, WA, USA). The center core of the transducer was removed for placement of a hydrophone allowing an overlap of their focal regions (Y-107, Sonic Concepts, WA, USA). The full setup of the system has been previously discussed elsewhere^13^. An acoustic pressure of 300 kPa was used for all procedures. The caudate, putamen, and the thalamus were targeted for the FUS procedures. The transducer was mounted on a stereotactic manipulator allowing 9-degrees of freedom for accurate targeting during anesthetized, lightly sedate and alert experiments (David Kopf Instruments, CA, USA). An initial 10-second sonication at 300 kPa without MB was acquired as a control for the real time cavitation monitoring to quantify background cavitation activity^51^. This was followed by administering a bolus of MB at a concentration of 2.5^8 ^MB/kg diluted in 3 ml of room temperature saline. The MB were administered at the onset of the 120-second sonication period.

Multiple anesthetized FUS procedures were performed on NHP A and Z before conducting the alert procedures (n = 13, n = 5 respectively). The full anesthetized procedure has been discussed elsewhere^13^. After the FUS procedure NHP were immediately transported to acquire MRI scans (3T, Philips Medical Systems, MA, USA) for verification of BBB opening and safety.

The lightly sedated FUS procedures were only conducted on NHP B. NHP B was given a low dose of ketamine (5 mg/kg) and placed into partial stereotactical positioning. The top canines were positioned to fit into a custom made bite bar which kept the NHP head steady and in a position similar to that as if it was in full stereotax positioning. Heart rate and blood pressure were monitored throughout the procedure. During the FUS procedure, the veterinary staff at NYSPI were present to observe if the procedure was having any gross negative effects on the body posture of the NHP or on the vitals. 6 hours after the procedure had finished, MRI scans were acquired to verify BBB opening and the safety of the procedure.

The setup of the alert experiment can be seen in Fig. 1. Multiple alert FUS procedures were conducted on both NHP A and Z (n = 9, n = 10 respectively). Initially the NHP were lightly sedated with ketamine (0.3 ml) for the placement of a catheter in the saphenous vein for IV delivery of the MB. NHP were then placed into the primate chair and head fixated. A pulse oximeter clip was placed on the ear ipsilateral to the placement of the transducer. The two positive EMG leads were placed on the temporalis muscle contralateral to the transducer with the ground placement ipsilateral to the transducer (MP150 Data Acquisition system, BIOPAC Systems Inc, CA, USA). Animals were allowed to work in the light and soundproof booth for an hour before beginning the FUS procedure. The FUS was applied at the onset of MB injection, which was through surgical tubing attached to the catheter extending from the work booth. After the FUS procedure finished NHP were allowed to work until satiated. MRI scans were acquired for each NHP 5 hours after the animal completed the behavioral tasks.

MRI scans for the aforementioned FUS procedures were as follows: T2-weighted and Susceptibility Weighted (SWI) scans were acquired to verify the safety of the FUS procedure. Following the acquisition of the T2-weighted and SWI scans, gadodiamide (Omniscan®, 573.66 DA, GE, Healthcare, Princeton, NY, USA, 0.2 ml/kg) was injected before acquiring contrast enhanced 3D T1-weighted images to verify BBB opening. Full specifications on the MRI acquisition have been previously reported^13^.

### Behavioral Testing

Behavioral control data were acquired with and without ketamine on separate days. For days when ketamine was administered, a dose of 0.3 ml ketamine was injected IM before testing to stay consistent when the FUS procedure was conducted. Non-ketamine control days verified the small dose of ketamine administered during the FUS procedure days had an effect on the behavioral results. The ketamine control days were utilized to find a regression curve over time to remove the effects of ketamine in the experimental days (Fig. 7b). NHP were placed in a primate chair for head fixation and positioning of the transducer. Both NHP were trained to respond to visual stimuli presented on a 20-inch color LCD touch panel display (NEC 2010X with 3M SC4 touch controller). The task utilized a reward magnitude bias (RMB) paradigm^40^,^41^. This task tested reaction time, touch error, and motivation. The visual stimulus on each trial was either a horizontal or vertical yellow bar randomly selected. The bars were of equal pixel area and intensity. The horizontal bar indicated a reward size of 5 drops, while the vertical bar indicated a reward size of 1 drop. An initial cue (either the horizontal or vertical bar) was presented randomly on either the left or right side of the screen. Once the NHP touched the cue, a secondary target, identical to the cue, appeared 4 cm to the left or right of the initial cue. After the NHP touched the target, the water reward was given based on the magnitude indicated by the stimuli. Reaction time to the cue and target was determined as the onset of the stimuli to the first touch registered by the touch panel. Touch error to the cue and target was determined by the distance between the center of the stimulus and the location where the NHP first touched the screen.

### Data Analysis

All data analysis pipelines were written in Matlab (MathWorks, MA, USA). Data sets analyzed were found to all be normally distributed.

T2-weighted and SWI scans were stereotactically aligned using fsl to determine if there was any hyper- or hypointense voxels in the targeted regions^48^. Post contrast T1-weighted scans were post processed to determine volume of BBB opening. The full pipeline of MRI processing has been discussed elsewhere^13^. Significance between the volume of opening data from the alert and anesthetized FUS procedures was determined with a 2-sided Wilcoxon rank-sum test.

Passive cavitation detection (PCD) data was acquired during all FUS procedures. The cavitation signal-processing pipeline has been previously implemented in our lab^27^. Significance between the PCD data from the alert and anesthetized FUS procedures was determined with a 2-sided Wilcoxon rank-sum test.

EMG of the temporalis muscle was recorded with surface (skin) electrodes. To determine heart rate, blood oxygenation was recorded with a pulse oximeter using an ear clip. Both signals were recorded at 2000 Hz. EMG signals were normalized by the mean to remove machine bias before applying a bandpass filter (100–300 Hz) to remove electrical noise and heart rate artifacts^53^. The EMG signal was full-wave rectified. A sliding window step detection algorithm was applied to locate muscle activity. Amplitudes within a maximum threshold recorded during the control were flagged to be voluntary movement. A peak-finding function was implemented with the pulse oximeter data to determine heart beats per minute (BPM). BPM was determined for three groups: pre, during and after the FUS procedure. A 1-way ANOVA was used to determine if there was significant variation in heart rate during the FUS procedure.

Behavioral data was divided by ketamine control days (days when no FUS procedure had occurred for at a minimum 5 days) and experimental days (days when the FUS procedure had occurred during behavioral testing). The control days were used to find normalization curves for both RT and TE data to reduce the effects of the ketamine from the experimental days (Fig. 7b). Data from the experimental days were divided into three groups, pre sonication, during sonication and post sonication. For analysis of the experimental data, trials only occurring 30 minutes before and after the sonication were selected. These trials were then normalized with the curve found with the ketamine control data. Variation between each groups was evaluated with 1-way ANOVAs (α = 0.05). Averages were compared across group for significant differences using a 2-sided Student’s t-test. Data was also subdivided by reward magnitude (high and low). The average differences (high – low) for these subgroups were compared for significant differences with a 2-sided Student’s t-test.

## Additional Information

**How to cite this article**: Downs, M. E. *et al.* Blood-Brain Barrier Opening in Behaving Non-Human Primates via Focused Ultrasound with Systemically Administered Microbubbles. *Sci. Rep.* **5**, 15076; doi: 10.1038/srep15076 (2015).

## Figures and Tables

**Table 1:** BBB opening per location for each NHP.

Target	NHP Z (Alert)	NHP A (Alert)	NHP Z(Anesthetized)	NHP A(Anesthetized)
Caudate	125.6 mm^3^(n = 1)	534.2 ± 261.2 mm^3^(n = 3/4)	N/A	N/A
Putamen	567.8 ± 251.6 mm^3^(n = 3/3)	417.3 ± 133.4 mm^3^(n = 4/4)	381.9 ± 245.6 mm^3^(n = 4/4)	453.9 ± 94.0 mm^3^(n = 9/12)
Thalamus	697.7 ± 181.8 mm^3^(n = 5/6)	352.1 mm^3^(n = 1/1)	382.5 mm^3^(n = 1)	400.4 mm^3^(n = 1)

Averages volumes and standard deviations here reflect only when BBB opening was successful. The n values signify successful BBB opening/Total FUS procedures at that location.
